# Clinical Features and Outcomes of Patients with Heart Failure and Advanced Chronic Kidney Disease

**DOI:** 10.3390/jcm14238508

**Published:** 2025-11-30

**Authors:** María Anguita-Gámez, Javier Herrera-Flores, Juan L. Bonilla-Palomas, Alejandro Recio-Mayoral, Rafael González-Manzanares, Juan C. Castillo Domínguez, José López-Aguilera, Javier Muñiz, Manuel Anguita-Sánchez

**Affiliations:** 1 Instituto Cardiovascular, Hospital Clínico San Carlos, 28040 Madrid, Spain; maria.anguita95@gmail.com; 2 UGC de Cardiología, Hospital Universitario Reina Sofía, Universidad de Córdoba, Instituto Maimónides de Investigación Biomédica de Córdoba (IMIBIC), 14004 Córdoba, Spain; javierherrera95@gmail.com (J.H.-F.); rafaelglezm@gmail.com (R.G.-M.); juanc.castillo.dominguez.sspa@juntadeandalucia.es (J.C.C.D.); jose.lopez.aguilera.sspa@juntadeandalucia.es (J.L.-A.); 3 Unidad de Cardiología, Hospital San Juan de la Cruz, 23400 Úbeda, Spain; juanl.bonilla.sspa@juntadeandalucia.es; 4 Hospital Universitario Virgen Macarena, 41009 Sevilla, Spain; jandrorm@hotmail.com; 5 Grupo de Investigación Cardiovascular, Departamento de Ciencias de la Salud e Instituto de Investigación Biomédica de A Coruña (INIBIC), Universidade da Coruña, 15006 A Coruña, Spain; proyectos@odds.es; 6 Centro de Investigación Biomédica en Red de Enfermedades Cardiovasculares (CIBER CV), 28029 Madrid, Spain

**Keywords:** heart failure, advanced chronic kidney renal disease, treatment, outcomes

## Abstract

**Objectives**: The aim was to evaluate the clinical features, management and 1-year outcomes in patients with heart failure (HF) and advanced chronic kidney disease (CKD) who were followed in specialized HF units in Spain. **Methods**: Data from the registry of the SEC-Excellent-HF quality program of the Spanish Society of Cardiology were analyzed. This registry included 1567 patients between 2019 and 2022 followed by 45 specialized HF units. Clinical features, treatment and 1-year rate of events (death and HF hospitalizations) were compared between the groups of advanced CKD (glomerular filtration rate <30 mL/minute/m^2^) and GFR ≥ 30 mL/min/m^2^. **Results**: 11.1% of patients had a GFR < 30 and 88.9% ≥ 30 mL/min/m^2^. The median LVEF was similar in groups with GFR < 30 and ≥30 mL/min/m^2^: 42% (IQR 30–58) versus 38% (IQR 29–54). Advanced CKD patients were older, had more severe HF (previous HF admissions in the last year, worse NYHA functional class and longer evolution time) and had higher prevalence of ischemic heart disease, diabetes mellitus, systemic hypertension, iron deficiency, anemia and hyponatremia. All drugs for HF, except for diuretics and potassium binders, were used in a lower proportion in patients with GFR < 30 mL/min/m^2^ (*p* < 0.001). One-year overall mortality (49.2 versus 13.7/100 patients-year; *p* < 0.001) and one-year HF hospitalizations rate (83.2 versus 30.7/100 patients-year; *p* < 0.001) were higher in the group of advanced CKD. **Conclusions**: In our study, patients with advanced CKD had different clinical characteristics, received indicated treatment in a lower proportion and had higher 1-year rates of death and HF admissions.

## 1. Introduction

Heart failure (HF) is a severe health problem because of its growing incidence and prevalence [1,2] and its poor prognosis and high incidence of severe events, including death [3,4]. Incidence of death and hospitalizations continue to be very high [3,4,5,6,7], in spite of the important advances made in recent years in its treatment.

Prevalence of chronic kidney disease (CKD) is increasing in patients with HF, with HF being an important cause of death and hospital admissions in these patients with renal failure [8]. About 17–50% of patients with HF have CKD, with rates of renal function impairment depending on the severity of CKD and patients’ age [9,10]. Prevalence and mortality of HF increases with worsening renal failure [8]. Renal impairment is an independent predictor for mortality and readmission rate in acute HF [8]. Advanced severe CKD worsens HF prognosis. Drug treatment that increases survival and decreases HF decompensations and hospital admissions is suboptimally prescribed in HF patients with severe kidney function impairment, despite current strong evidence showing the benefits of many of these drugs (renin–angiotensin–aldosterone inhibitors, β-blockers, neprilysin inhibitors and mineralocorticoid receptor antagonists) [11,12,13,14], mainly due to concerns about renal function worsening and hyperkalemia [11,12]. There is growing evidence for the use of sodium–glucose co-transporter 2 inhibitors [15,16,17] in the management of patients with HF and CKD, but few studies have included patients with advanced CKD and patients on dialysis, limiting the knowledge about its efficacy and safety in this condition. This problem will continue to grow, because of the increased survival in these patients, and it is likely that the number of patients with both problems will continue to increase.

In addition, there is not much knowledge regarding patients with more severe renal dysfunction, with GFR < 30 mL/min/m^2^, since clinical drug trials usually exclude these patients, and, moreover, real-life registries do not separately analyze the degree of renal dysfunction. Thus, the aims of our study were to analyze the prevalence of advanced CKD in a contemporary survey of patients with HF in Spain and to compare clinical characteristics, treatment and 1-year outcomes (overall death and HF decompensations rates) in these two groups of HF patients.

### 1.1. Methods

The SEC-Excellent-HF Registry is an ongoing, prospective, multicentric, observational study of patients with HF followed in 45 HF units participating in the SEC-Excellent-HF quality program of the Spanish Society of Cardiology. The design and logistics of this program have recently been published [18]. The study met all requirements of the Declaration of Helsinki for research involving human subjects and was approved by the local ethics committees of the participating centers. All patients signed the informed consent document. Each unit included all HF patients in two 1-month cutoffs (March and October) in 2019 to 2022, with 1716 patients included in the registry in this period. Patients completed the 1-year follow-up in December 2023. Of the 1761 patients included, no information was available at 1 year of follow-up in 149 cases (8.7%), so the final analysis was performed on 1567 patients. Follow-up losses were evenly distributed in both GFR groups.

### 1.2. Inclusion Criteria and Follow-Up

We included inpatients and outpatients with a recent hospital admission (within the previous 3 months) with a primary diagnosis of HF. There were no specific exclusion criteria, other than patients age less than 18 years. Left ventricular ejection fraction was determined at the baseline visit, and HF reduced (HFrEF), mildly reduced (HFmrEF) or preserved (HFpEF) were defined according to the ESC guidelines criteria [7]. Advanced CKD was defined by an estimated CKD-EPI GFR < 30 mL/minute/m^2^ value at discharge after an HF admission or at the inclusion visit in outpatients. Table 1 and Table 2 show the main clinical and treatment characteristics of our patients at the baseline. A follow-up visit was performed 1 year after inclusion for all patients to obtain information about clinical outcomes (death, HF hospitalizations and decompensations of HF without hospitalization). Death was identified in the clinical history or after a phone call if the patient did not attend the scheduled visit. A hospital admission of more than 24 h with the diagnosis of HF was considered as HF hospitalization [7] Need for intravenous of diuretics or inotropic agent administration or an emergency room visit, without hospital admission, were considered as HF decompensation without hospitalization. Patients who died during the hospital admission that led to inclusion in the study were excluded from the follow-up analysis. All patients were followed-up and treated according to the criteria of their physicians, without any prespecified intervention as part of the registry protocol.

### 1.3. Statistical Analysis

Qualitative variables are expressed as proportions and continuous variables are presented as median [IQR]. The chi-square test or Fisher’s exact test, in the case of qualitative variables, and the Kruskal–Wallis test for non-parametric continuous variables were used. to compare differences between the two groups of GFR < or ≥30 mL/min/m^2^. One-year incidences of death, HF admission, HF decompensation without hospital admission and that of the combined event of death/HF admission were calculated and expressed as incidence rates per 100 patient-years. The incidences of the different events were compared between the two subgroups of patients as relative risks (Table 3). A multivariate Cox regression analysis was carried out to determine features independently associated with mortality, and a negative binomial regression model was used to analyze variables associated with HF admissions and decompensations, to correct data overdispersion. Models were performed with a backward procedure, using a value of *p* < 0.05 to remain in the final model. Variables that were significant in the univariate analysis (*p* value < 0.10) were included in the multivariate model. The STATA 12.0 statistical package was used for analysis.

## 2. Results

### 2.1. Baseline Features and Treatment

Of the 1567 patients, 11.1% had a glomerular filtration rate < 30 mL/min. Patients with advanced CKD were older and had a higher prevalence of hypertension, diabetes mellitus, coronary heart disease, malnutrition, anemia and iron deficiency. There was no difference in the proportion of women. The left ventricular ejection fraction was similar, as was the proportion of HFrEF and HFpEF (Table 1). Etiology was more frequently of ischemic and hypertensive origin in the group with GFR < 30 mL/min/m^2^.

**Figure 1:**
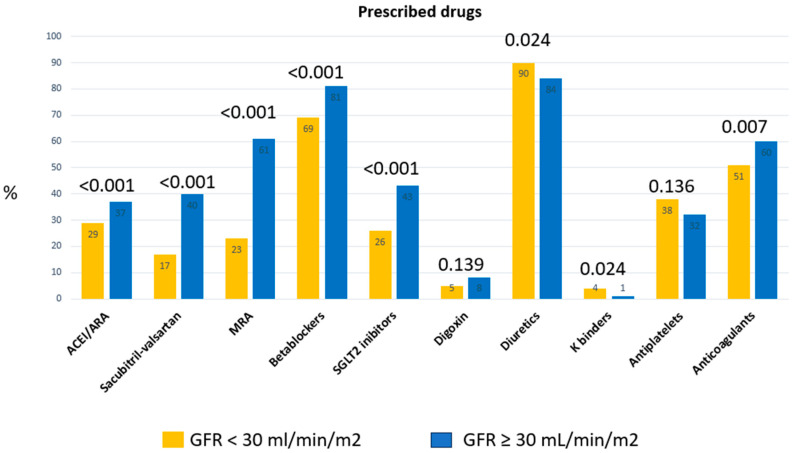
Prescribed drugs in the 2 subgroups of the glomerular filtration rate. ACEI: angiotensin converting enzyme inhibitors. ARB: angiotensin receptor blockers. MRA: mineral receptor antagonists. K: potassium.

Patients with GFR < 30 mL/min/m^2^ received in a lower proportion all drugs with a feasible prognostic effect (Table 2), including betablockers, sacubitril-valsartan, MRA and SGLT2 inhibitors. They also received a smaller proportion of direct oral anticoagulants. They were taking diuretics and potassium chelators in a higher proportion than those in the group with less impaired renal function. These results were similar when the analysis was limited to patients with HFrEF. Patients with HFrEF and GFR < 30 mL/min/m^2^ received sacubitril-valsartan. They received MRA, betablockers and SGLT2 inhibitors in a significantly lower proportion than those with HFrEF and GFR ≥ 30 mL/min/m^2^ (Table 3). In the overall series, there were no differences in the use of AIDs and CRT (Table 1), and they underwent a lower proportion of cardiac rehabilitation programs (Table 2). Figure 1 graphically shows the differences in the prescribed drugs between the two groups of patients.

### 2.2. Incidence of 1-Year Events

Table 4 shows the incidence rates of the different events; 32 of the 1239 patients included during an HF hospitalization episode died during admission (in-hospital mortality 2.6%). During follow-up, 241 deaths, 170 decompensations of HF without hospitalization and 434 admissions for HF were identified. In the overall series, the mortality rate was 16.9/100 patient-years (95% CI: 14.9–19.1) and that of HF decompensation without admission was 11.9 (10.2–13.8), HF admissions was 30.4 (27.7–33.4) and overall decompensations, including admissions, was 42.4 (39.1–45.9) per 100 patient-years.

**Figure 2:**
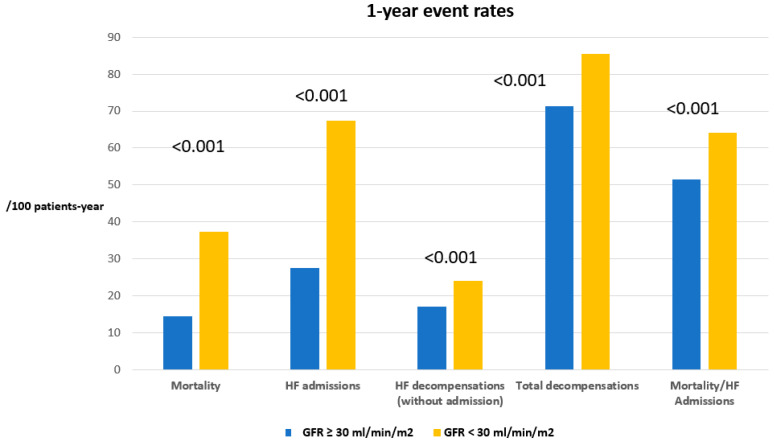
Event rates at 1 year, in the two subgroups of the glomerular filtration rate. HF: heart failure. GFR: glomerular filtration rate.

Mortality, HF hospitalization rate, incidence of HF decompensation and total decompensation rate at 1 year were statistically higher in the group with GFR < 30 mL/min/m^2^ (*p* < 0.001 for all events; Table 4). Patients with GFR ≥ 30 mL/min/m^2^ had a 62% lower mortality, a 56% lower HF admission rate and a 55% lower HF decompensation rate (Table 4). Figure 2 graphically shows the differences in the event incidence between the two groups of patients.

Most deaths, 75.4% of all deaths, were of cardiovascular origin, without differences between both groups (78.1% in patients with GFR < 30 mil/min/m^2^ versus 74.9% in the other group). Deaths were due to HF progression in 54.6% and 50.3%, to acute myocardial infarction in 9.6% and 10.7%, to stroke in 7.8% and 9.6% and to sudden death in 6.1% and 7.5%, respectively, in both groups. In the multivariate analysis, advanced CKD was significantly associated with all events. The HR for mortality was 1.62 (95% CI: 1.23–2.13). The incidence risk ratio for HF hospitalizations was 1.44 (95% CI: 1.09–1.92), and for HF decompensations it was 1.58 (95% CI: 1.23–2.01) (*p* < 0.001 for all events). The sensitivity analysis confirmed these results. Other features independently associated with mortality were age (HR 1.03; 95% CI 1.01–1.04), ischemic etiology (HR 1.47; 95% CI 1.13–1.91), HF admissions in the previous year (HR 1.62; 95% CI 1.23–2.13), chronic obstructive pulmonary disease (HR 1.43; 95% CI 1.03–1.97), cancer (HR 1.78; 95% CI 1.18–2.68) and malnutrition (HR 1.76; 95% CI 1.16–2.67). Besides advanced CKD, HF admission in the previous year (HR 2.33; 95% CI 1.25–2.77, ischemic etiology (HR 1.60; 95% CI 1.20–2.14), atrial fibrillation (HR 1.82; 95% CI 1.37–2.41), chronic obstructive pulmonary disease (HR 1.50; 95% CI 1.08–2.08) and anemia (HR 1.36; 95% CI 1.02–1.81) also were independently associated with HF admissions.

## 3. Discussion

CKD is showing an increased prevalence in HF patients, and HF is commonly diagnosed in patients with CKD, with this magnitude depending on the severity of the CKD and patient age [9,10]. The prognosis of HF is worse in patients with severe impairment of renal function [8], and kidney dysfunction is independently associated with higher mortality of patients with acute HF [8]. But there are few studies that have specifically analyzed the impact of advanced CKD, with GFR < 30 mL/min/m^2^, on the prognosis of HF, as well as on its pharmacological treatment, because HF registries do not distinguish the degree of renal function impairment [3,19,20,21,22], and clinical trials exclude these patients [14,15,16,17,23,24]. Our study specifically analyzes the results in HF and advanced CKD, comparing clinical features, treatment and outcomes between patients with GFR < or ≥30 mL/min/m^2^. Prevalence of advanced CKD was high, 11.1% in our study, without differences according to the type of HF (HFrEF, HFmrEF or HFpEF). As shown in Table 3, 10.4% of patients with HFrEF had a GFR < 30 mL/min/m^2^, a proportion similar to that found in the overall series.

Other main results on our study indicate that patients with HF and advanced CKD receive a significantly lower proportion of the drugs with a favorable prognostic effect in HF (Table 2 and Table 3) and that the incidence of serious events at one year of follow-up (death, admissions and HF decompensations) is 2 to 3 times higher in this group, as shown in Table 4. Patients with advanced CKD were older and had a higher prevalence of hypertension, diabetes mellitus, coronary heart disease, malnutrition, anemia and iron deficiency, and etiology was more frequently of ischemic and hypertensive origin in the group with GFR < 30 mL/min/m^2^. This higher risk profile and prevalence of severe comorbidities may influence this worse prognosis, but in the multivariate study, a GFR < 30 mL/min/m^2^ was associated with a higher total mortality and incidence of HF admissions and decompensations at one year. Advanced CKD was strongly associated with such events, with a hazard ratio of 1.63 for mortality, 1.44 for HF admissions and 1.58 for HF decompensation at 1 year. These figures are higher than those shown by several recent European registries, which only analyze globally the impact of the existence of renal dysfunction, defined by a GFR < 90 or <60 mL/min/m^2^, without separating the degree of severity of renal failure [3,4,19,20,21,22].

Once the degree of renal function impairment has already reached a high level of severity, the prognosis of patients worsens, and it is difficult to reduce the incidence of events. Therefore, it would be important to initiate drug treatment with favorable prognostic effect in patients with HF, especially with HFrEF, but also in HFpEF and HFmrEF, in less advanced stages of CKD, I to III, when these treatments can be introduced and optimized with certain safety [13,14,15,16,17]. There are several mechanisms that can influence the interaction between the drugs used for the treatment of HF and renal function, either favorably or unfavorably, such as neurohormonal activation, altered potassium homeostasis and drug metabolism changes [8]. In any case, even in patients with GFR < 30 mL/min/m^2^, pharmacological treatment should be introduced, prioritizing drugs with lower risk of renal function deterioration, such as betablockers and SGLT2 inhibitors, and even sacubitril-valsartan, closely monitoring renal function and serum potassium, to improve the prognosis of these patients. It is difficult to speculate about the effects of MRA and SGLT2 inhibitors in patients with HF and severe CKD, since MRA can affect kidney function and cause hyperkalemia, and there are few studies on the effect of SGLT2 inhibitors in these patients. Probably because of this, the proportion of patients in the group with GFR < 30 is very low, so reliable conclusions cannot be drawn. Specific studies need to be conducted in this regard. It is possible that the introduction of new MRA, such as finerenone, with a greater protective effect on renal function impairment [24,25], may help to improve this problem.

In a recent meta-analysis [26], the authors have evaluated the role of different drugs in various subgroups of patients with HF, including CKD. Three of them significantly reduce cardiovascular mortality/hospitalizations for HF among patients with eGFR <60 mL/min/m^2^ compared to placebo: sacubitril/valsartan, SGLT2 inhibitors (mainly dapagliflozin) and vericiguat. Although the study does not specifically include only patients with GFR < 30 mL/min/m^2^, the beneficial effects of dapagliflozin also may extend to patients with lower GFR.

## 4. Limitations and Conclusions

Our study has several limitations. First, it is an observational study but in the same way as most registries and surveys. Although kidney function may have varied throughout the study, the criterion for considering that a patient had a GFR < 30 mL/min/m^2^ was the value at study inclusion, either at discharge in the patients included during a decompensation episode or at the baseline visit in outpatients. Thus, it is not possible to differentiate the impact of an acute deterioration of kidney function, although the GFR one year after inclusion was still < 30 mL/min/m^2^ in most cases. The value of proteinuria was also not analyzed, as this data was missing in many patients. Another limitation is the lack of use of drugs that we know today have a favorable prognostic effect, recommended by clinical practice guidelines, but whose evidence was not available at the time of inclusion of patients in the registry, such as SGLT2 inhibitors. However, it covers a very recent period and has the strength of the mandatory participation of all the centers that received the quality accreditation of our program, which reduces the inclusion bias. Since data are collected only in specialized HF units, this fact can limit the generalizability of the results. With these limitations in mind, it can be concluded from our results that patients with HF and advanced CKD receive a significantly lower proportion of drugs with a favorable prognostic effect in HF and that the incidence of serious events at one year of follow-up (death, admissions and HF decompensations) was 2 to 3 times that in patients with a less severe degree of renal dysfunction. A greater effort in the prevention of renal damage and a better optimization of treatment, including new drugs with nephroprotective effects, may help to improve this unfavorable prognosis.

## Figures and Tables

**Table 1:** Demographic characteristics, risk factors, comorbidities and history of heart failure in patients with glomerular filtration rate < or % ≥30 mL/min/m^2^.

	All n = 1567	GFR < 30 mL/min/m^2^ n = 174 (11.1%)	GFR ≥ 30 mL/min/m^2^ n = 1393 (88.9%)	*p* Value
**Age (years)**	71 (63–87)	76 (63–88)	70 (60–85)	<0.001
**Female sex**	37.1%	39.7%	36.4%	0.741
**Hypertension**	72.9%	85.5%	71.1%	<0.001
**Diabetes mellitus**	43.8%	59.9%	41.1%	<0.001
**Coronary artery disease**	31.6%	40.8%	28.9%	0.001
**Coronary revascularization**	31.3%	37.4%	30.7%	0.013
**Stroke**	9.8%	11.1%	9.6%	0.535
**Malnutrition**	4.5%	8.2%	4.1%	0.016
**Anemia**	34.4%	63.6%	30.6%	<0.001
**Cancer**	6.5%	8.1%	6.1%	0.292
**Atrial fibrillation**	52.5%	49.5%	53.5%	0.015
**Permanent atrial fibrillation**	28.4%	23.2%	27.6%	<0.001
**Chronic pulmonary obstructive disease**	17.0%	16.3%	17.4%	0.644
**Ferropenia**	33.8%	42.9%	32.6%	0.007
**LVEF (%)**	39 (29–58)	42 (30–58)	38 (29–54)	0.063
**HFrEF**	55.5%	56.7%	52.8%	0.246
**De novo HF**	49.5%	35.7%	52.4%	<0.001
**HF admissions within the previous year**	33.5%	48.3%	31.3%	<0.001
**Previous CRT**	7.9%	8.2%	7.8%	0.730
**Previous AID**	14.5%	12.6%	14.8%	0.735
**HF etiology**				<0.001
**-** **Ischemic**	31.6%	39.1%	30.5%	
**-** **Hypertensive**	8.2%	13.8%	6.9%	
**-** **Idiopathic**	13.7%	5.7%	15.2%	
**-** **Valvular**	17.4%	16.1%	17.6%	
**-** **Tachycardiomyopathy**	12.3%	7.5%	13.5%	
**NHYA III-IV class**	60.8%	77.3%	58.5%	<0.001
**Left bundle branch block**	23.7%	26.6%	23.7%	0.409
**Body mass index (kg/m^2^)**	28 (24–33)	28 (24–33)	28 (25–33)	0.643
**Glomerular filtration rate (mL/min/m^2^)**	59 (23–75)	27 (21–29)	65 (38–75)	<0.001
**Hemoglobin (g/dL)**	13 (9–14)	11 (9–12)	13 (9–14)	<0.001
**NTproBNP (ng/mL)**	4543 (2653–9631)	9665 (4265–17,642)	3286 (2364–6498)	<0.001

GFR: glomerular filtration rate. HF: heart failure. LVEF: left ventricular ejection fraction. HFrEF: heart failure with reduced ejection fraction. CRT: cardiac resynchronization therapy. AID: automatic implantable cardioverter.

**Table 2:** Treatment at the inclusion visit in the registry for patients with glomerular filtration rate < or % ≥30 mL/min/m^2^.

	All n = 1567	GFR < 30 mL/min/m^2^ n = 174 (11.1%)	GFR ≥ 30 mL/min/m^2^ n = 1393 (88.9%)	*p* Value
**ACEI/ARB**	36.6%	29.3%	37.2%	<0.001
**Sacubitril-valsartan**	38.0%	17.2%	40.6%	<0.001
**Mineral receptor antagonists**	56.6%	22.9%	61.1%	<0.001
**Betablockers**	80.0%	69.5%	81.3%	<0.001
**SGLT2 inhibitors**	40.1%	26.6%	42.7%	<0.001
**Diuretics**	84.9%	90.2%	83.6%	0.024
**Digoxin**	8.2%	5.2%	10.3%	0.139
**Ivabradine**	9.1%	7.5%	9.4%	0.404
**Antiplatelets**	32.9%	37.8%	32.1%	0.136
**Oral anticoagulants**	57.8%	50.6%	59.4%	0.007
**Direct anticoagulants**	39.8%	29.3%	41.7%	<0.001
**Potassium chelators**	1.9%	4.1%	1.6%	0.024
**Cardiac rehabilitation program**	9.6%	5.2%	11.1%	0.020

GFR: glomerular filtration rate. ACEI: angiotensin converting enzyme inhibitors. ARB: angiotensin receptor blockers.

**Table 3:** Pharmacological treatment at the inclusion visit in the subgroup of patients with heart failure with reduced left ventricular ejection fraction according to glomerular filtration rate < or ≥30 mL/min/m^2^.

	GFR < 30 mL/min/m^2^ n = 91 (10.4%)	GFR ≥ 30 mL/min/m^2^ n = 779 (89.6%)	*p* Value
**ACEI/ARB**	19.5%	29.2%	0.063
**Sacubitril-valsartan**	30.5%	59.2%	<0.001
**Mineral receptor antagonists**	31.7%	77.8%	<0.001
**Betablockers**	76.8%	89.5%	0.001
**SGLT2 inhibitors**	34.1%	52.9%	0.001
**Diuretics**	91.4%	81.7%	0.031
**Digoxin**	7.3%	8.8%	0.837
**Ivabradine**	14.6%	14.1%	0.888
**Antiplatelets**	43.9%	37.6%	0.267
**Oral anticoagulants**	47.6%	52.5%	0.005
**Direct anticoagulants**	21.9%	37.4%	0.005
**Potassium chelators**	3.6%	1.9%	0.405

GFR: glomerular filtration rate. ACEI: angiotensin converting enzyme inhibitors. ARB: angiotensin receptor blockers.

**Table 4:** Overall incidence rate of the events of interest and according to glomerular filtration rate < or ≥30 mL/min/m^2^. Rate expressed in events/100 patient-years.

	Event Number	Incidence Rate	95% CI		Relative Risk	95% CI		*p* Value
**Mortality**								
**GFR < 30**	51	37.26	28.32	49.03	Reference			
**FG ≥ 30**	172	14.44	12.44	16.77	0.38	0.28	0.53	<0.001
**HF hospitalization**								
**GFR < 30**	84	61.37	49.56	76.00	Reference			
**GFR ≥ 30**	328	27.55	24.72	30.69	0.44	0.35	0.57	<0.001
**Death/HF hospitalization**								
**GFR < 30**	77	64.26	51.40	80.35	Reference			
**GFR ≥ 30**	328	29.89	26.82	33.30	0.46	0.36	0.59	<0.001
**HF decompensation without hospitalization**								
**GFR < 30**	33	24.11	17.14	33.91	Reference			
**GFR ≥ 30**	131	11.00	9.27	13.06	0.45	0.31	0.66	<0.001
**All HF decompensation**								
**GFR < 30**	117	85.48	71.31	102.46	Reference			
**GFR ≥ 30**	459	38.55	35.18	42.24	0.45	0.36	0.55	<0.001

CI: confidence interval. GFR: glomerular filtration rate (mL/min/m^2^).

## Data Availability

The original contributions presented in this study are included in the article. Further inquiries can be directed to the corresponding author.
